# Pregnant People-Infant Linkage for Surveillance

**DOI:** 10.23889/ijpds.v9i5.2637

**Published:** 2024-09-18

**Authors:** Nicole Satherley, Andrew Sporle

**Affiliations:** 1 iNZight Analytics; 2 University of Auckland

## Objective

The COVID-19 pandemic has exposed numerous social inequities in health outcomes across nations, despite early warnings of their potential. Poorer outcomes for ethnic minorities, and to a lesser extent Indigenous peoples, have been widely reported, and may be explained in part by household conditions and poorer socio-economic circumstances. This study aimed to identify the effects of diverse individual and social conditions on COVID-19 outcomes within the New Zealand population.

## Method

We conducted a whole population analysis of the association between individual (e.g. ethnicity, disability status), household (e.g. household composition), and socio-economic (e.g. crowding, housing quality, income) factors and four COVID-19 health outcomes – infection, hospitalisation, mortality, and vaccination status. We constructed variables of interest from linked administrative data in New Zealand’s Integrated Data Infrastructure, and examined associations within the 2018 Census usually resident population (analysis Ns = 518,571 - 3,216,696).

## Results

Analyses showed that Māori (the Indigenous people of New Zealand) and Pacific peoples, an ethnic minority, experienced worse outcomes than other New Zealanders across all COVID-19 outcomes. These ethnic group differences were persistent, yet reduced, when accounting for household and socio-economic variables. Factors including disability status, high housing mobility, poor quality housing, and household crowding were also widely predictive of worse outcomes.

## Conclusion

Ethnic inequity in a range of COVID-19 outcomes is present in a country where the overall impacts of the pandemic were relatively limited. Individual, household, and socio-economic factors are associated with diverse COVID-19 outcomes, and policy amenable factors may help explain the presence of ethnic inequities.

